# When rare meets rarer: constrictive pericarditis in Erdheim–Chester disease—a case report

**DOI:** 10.1093/ehjcr/ytag619

**Published:** 2026-08-30

**Authors:** Maria Dimova-Mileva, Iliyan Kanchev, Elena Marinova, Georgi Bachvarov, Anita Dimitrova

**Affiliations:** Medical University of Varna, 55, Marin Drinov, str., Varna, 9002, Bulgaria; Clinic of Internal Medicine, University Hospital ‘St. Marina’, 1, Hr. Smirnenski, Bul., Varna 9010, Bulgaria; Medical University of Varna, 55, Marin Drinov, str., Varna, 9002, Bulgaria; Clinic of Internal Medicine, University Hospital ‘St. Marina’, 1, Hr. Smirnenski, Bul., Varna 9010, Bulgaria; Medical University of Varna, 55, Marin Drinov, str., Varna, 9002, Bulgaria; Clinic of Internal Medicine, University Hospital ‘St. Marina’, 1, Hr. Smirnenski, Bul., Varna 9010, Bulgaria; Medical University of Varna, 55, Marin Drinov, str., Varna, 9002, Bulgaria; Clinic of Cardiac Surgery, University Hospital ‘St. Marina’, 1, Hr. Smirnenski, Bul., Varna 9010, Bulgaria; Medical University of Varna, 55, Marin Drinov, str., Varna, 9002, Bulgaria; Clinic of General and Clinical Pathology, University Hospital ‘St. Marina’, 1, Hr. Smirnenski, Bul., Varna 9010, Bulgaria

**Keywords:** Erdheim–Chester disease, Case report, Constrictive pericarditis, Cardiovascular involvement, Multimodality imaging, Clinicopathological correlation

## Abstract

**Background:**

Erdheim–Chester disease (ECD) is a rare clonal non-Langerhans cell histiocytosis driven predominantly by MAPK pathway alterations. Although cardiovascular involvement is frequent, it is usually subclinical, while constrictive pericarditis is exceptionally rare.

**Case summary:**

A 57-year-old man with multisystem ECD presented with signs of congestive heart failure. Multimodality imaging demonstrated extensive cardiovascular involvement, with marked pericardial thickening and haemodynamic features of constrictive pericarditis on echocardiography, diffuse late gadolinium pericardial enhancement on cardiac magnetic resonance, and circumferential periarterial infiltration on computed tomography angiography. Despite intensive medical therapy resulting in temporary haemodynamic improvement, persistent constrictive physiology prompted urgent pericardiectomy. Intraoperatively, the pericardium was diffusely thickened and densely adherent to the epicardium. The post-operative course was complicated by refractory cardiogenic shock and multiorgan failure, leading to the patient’s death. Autopsy revealed extensive histiocytic infiltration of the pericardium and myocardium, demonstrating that extensive cardiovascular involvement in ECD, rather than isolated pericardial fibrosis, was causative for the constrictive physiology.

**Discussion:**

This case illustrates the potentially devastating consequences of cardiovascular involvement in ECD. It suggests that once extensive myocardial infiltration by histiocytes additional to pericardial fibrosis has evolved, pericardiectomy alone may be insufficient to relieve the constrictive physiology and alter clinical course.

Learning points

- Constrictive pericarditis may be the dominant manifestation of cardiovascular involvement in Erdheim–Chester disease.
- Pericardiectomy may be effective for constrictive pericarditis in selected patients with Erdheim–Chester disease, particularly in the absence of extensive myocardial infiltration.

## Introduction

Erdheim–Chester disease (ECD) is a rare multisystemic disorder belonging to the group of non-Langerhans cell histiocytoses, included in the 2016 World Health Organization classification of haematopoietic tumours. The disease is driven predominantly by constitutive activation of the MAPK signalling pathway, with BRAF mutations identified in ∼57%–70% of patients.^1^ The clinical presentation is highly heterogeneous and depends on the extent and distribution of organ involvement. The introduction of targeted therapies has substantially improved the prognosis of ECD over the past two decades.

The disorder is exceptionally rare, with ∼1500 cases reported worldwide to date.^1^ Cardiovascular involvement, although being common on imaging examinations, is frequently clinically silent. Typical findings include peri-aortic and peri-coronary soft-tissue infiltration, myocardial infiltration, right atrioventricular pseudotumours, and pericardial disease.^1–4^ Although pericardial thickening and effusion are relatively common, progression to constrictive pericarditis is casuistic, with only a few cases reported in the literature.^5–9^

We present a case of a patient with advanced multisystem ECD and clinical findings of heart failure due to constrictive pericarditis. Multimodality imaging, surgical findings, and autopsy correlation demonstrated that constrictive physiology represented diffuse cardiovascular histiocytic infiltration rather than isolated pericardial disease, highlighting the challenges of therapeutic decision-making in advanced cardiac ECD.

## Summary figure

**Figure:**
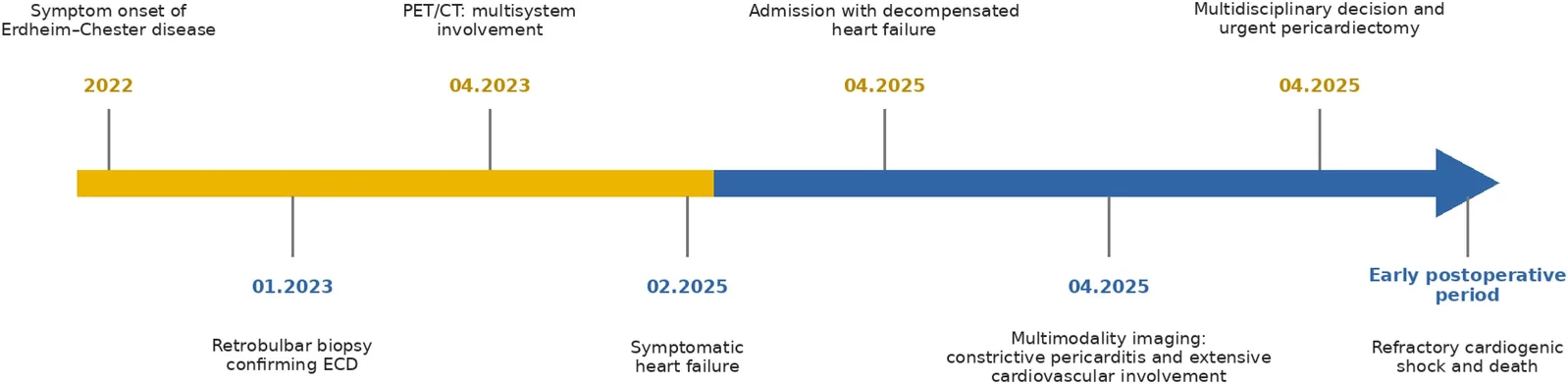


## Case presentation

A 57-year-old man with ECD was admitted because of progressive dyspnoea, severe bilateral lower-extremity oedema associated with painful exudative skin lesions, and worsening leg pain.

The diagnosis of ECD was established 3 years earlier at another institution following biopsy of a retrobulbar mass causing bilateral exophthalmos. Molecular analysis showed no BRAF mutation. Staging positron emission tomography/computed tomography (PET/CT) demonstrated extensive multisystem involvement affecting the retroperitoneum, kidneys, testes, skeleton, pituitary gland, and cardiovascular system, including both the pericardium and myocardium. The patient had been diagnosed with primary hypothyroidism and central diabetes insipidus 2 years before the current hospital admission. Chronic medications included hormone replacement therapy—desmopressin and levothyroxine, loop diuretic, SGLT2 inhibitor, and ACE inhibitor, prescribed for clinical signs of heart failure 3 months earlier. Glucocorticoids were initiated at the time of the ECD diagnosis, but disease-specific therapy was never administered.

On admission, the patient appeared acutely ill, with clinical signs of decompensated congestive heart failure. Blood pressure was 80/60 mmHg, with elevated jugular venous pressure, diminished breath sounds, bilateral basal crackles, and muffled heart sounds. Marked lower-extremity oedema extending to the thighs was associated with cutaneous erosions covered by haemorrhagic crusts and purulent discharge. Bilateral exophthalmos and xanthelasma of the lower eyelids were also present.

**Figure 1:**
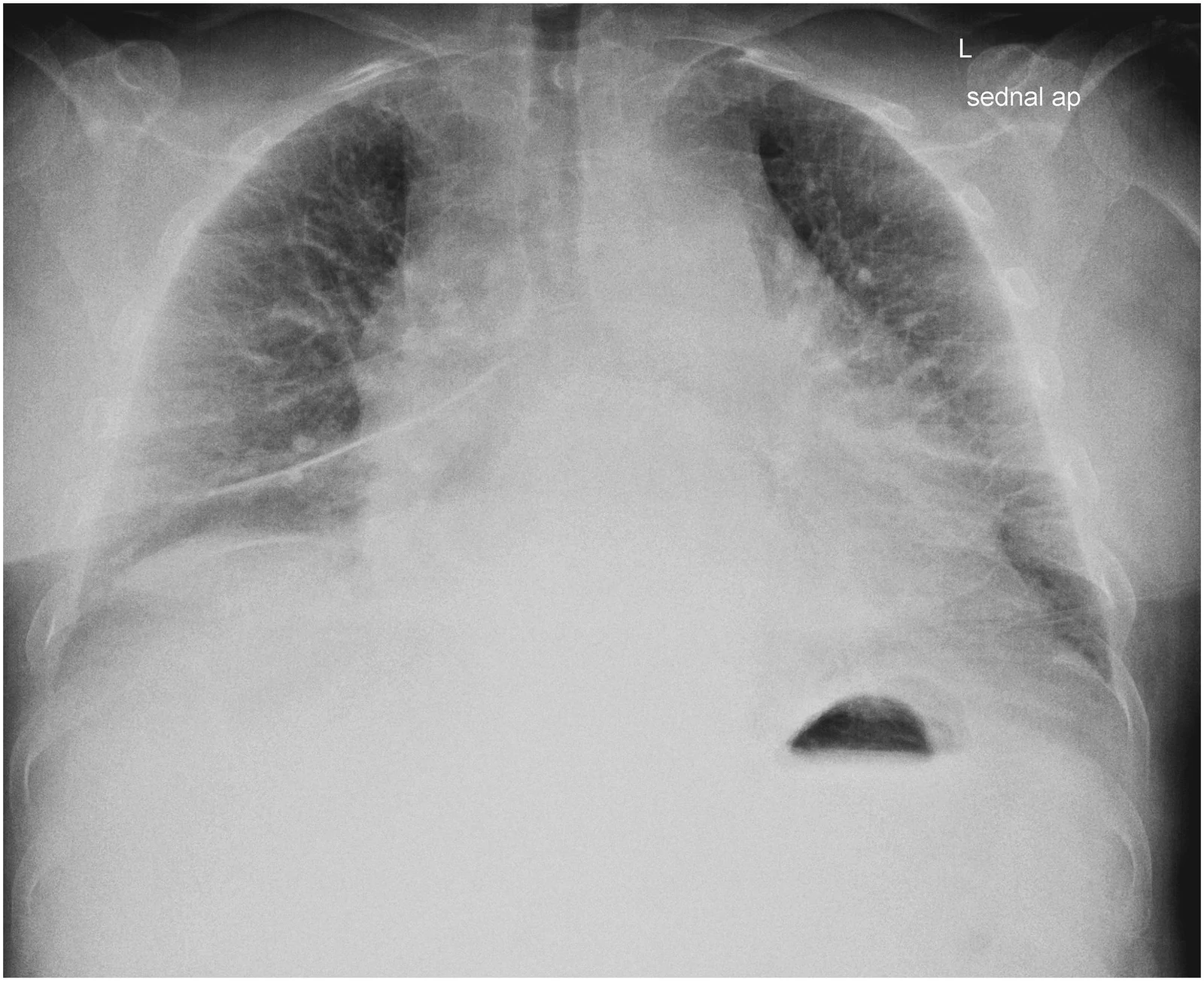
Chest radiography. Chest radiography demonstrated an enlarged cardiac silhouette associated with interstitial pulmonary congestion and bilateral pleural effusions.

Electrocardiography demonstrated sinus rhythm with low QRS voltage in the limb leads (<0.5 mV). Chest radiography revealed cardiomegaly, bilateral pleural effusions, and interstitial pulmonary congestion (*Figure 1*). Laboratory investigations showed elevated inflammatory markers, including a C-reactive protein level of 81 mg/L (reference range, 0–5 mg/L) and leukocytosis with absolute neutrophilia, together with a markedly elevated NT-proBNP concentration of 14 058 pg/mL (reference range, 0–800 pg/mL).

**Figure 2:**
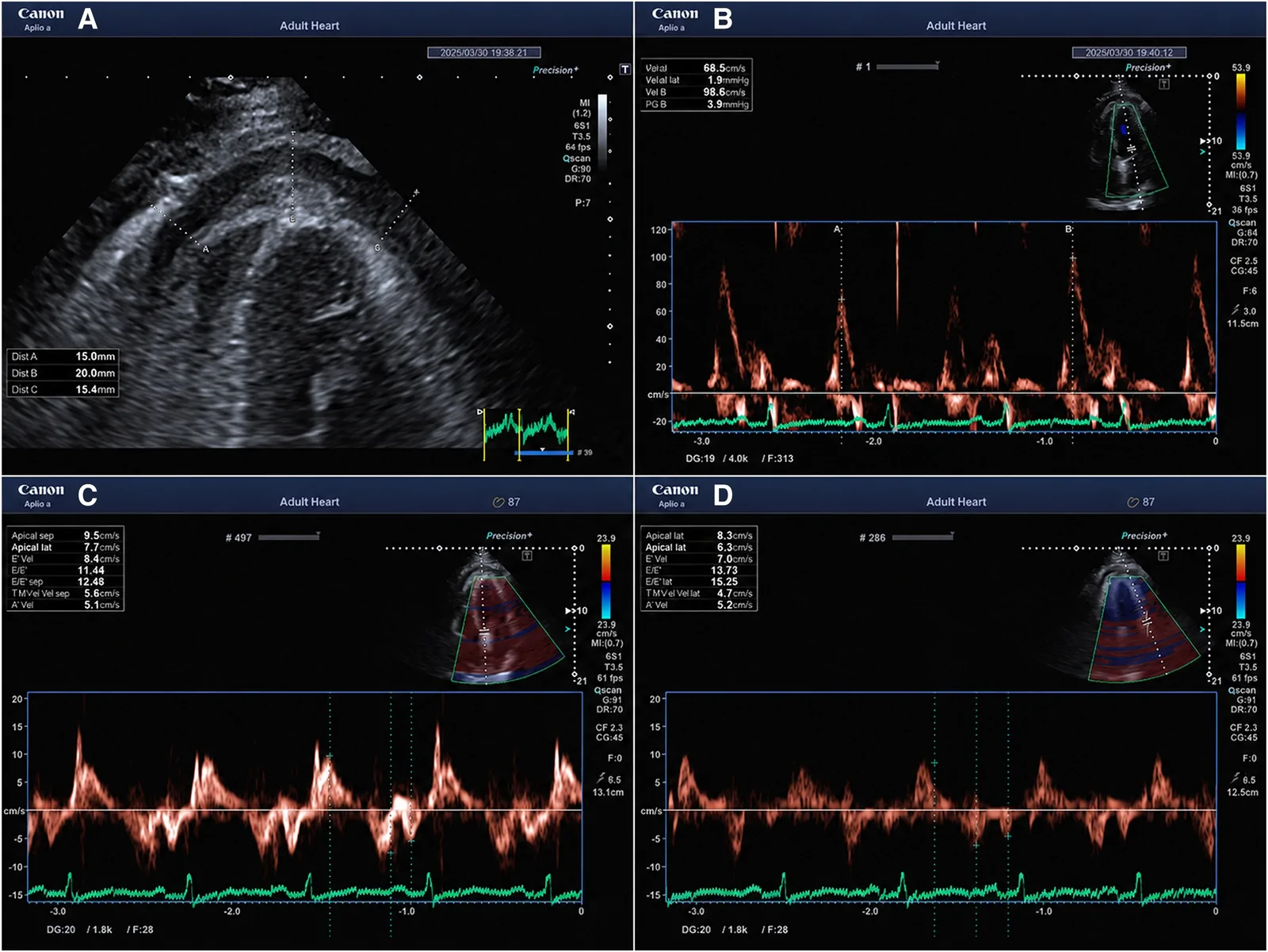
Echocardiographic findings. Transthoracic echocardiography demonstrated marked pericardial thickening of up to 20 mm (*A*), a transmitral E/A ratio > 2 with ∼30% respiratory variation in E-wave velocity (*B*), and markedly reduced lateral mitral annular E′ velocity, consistent with annulus reversus (*C* and *D*).

Transthoracic echocardiography demonstrated preserved left ventricular systolic function and mild left atrial enlargement. The most striking finding was marked pericardial thickening, reaching a maximum thickness of 20 mm in some segments (*Figure 2A*). Classical haemodynamic features of constrictive pericarditis were present, including a transmitral E/A ratio of 2.1, ∼30% respiratory variation in transmitral E-wave velocity (*Figure 2B*), markedly reduced lateral mitral annular early diastolic velocity (annulus reversus) (*Figure 2C*  and  *D*), and paradoxical interventricular septal motion (septal bounce). Based on these findings, constrictive pericarditis was diagnosed.

**Figure 3:**
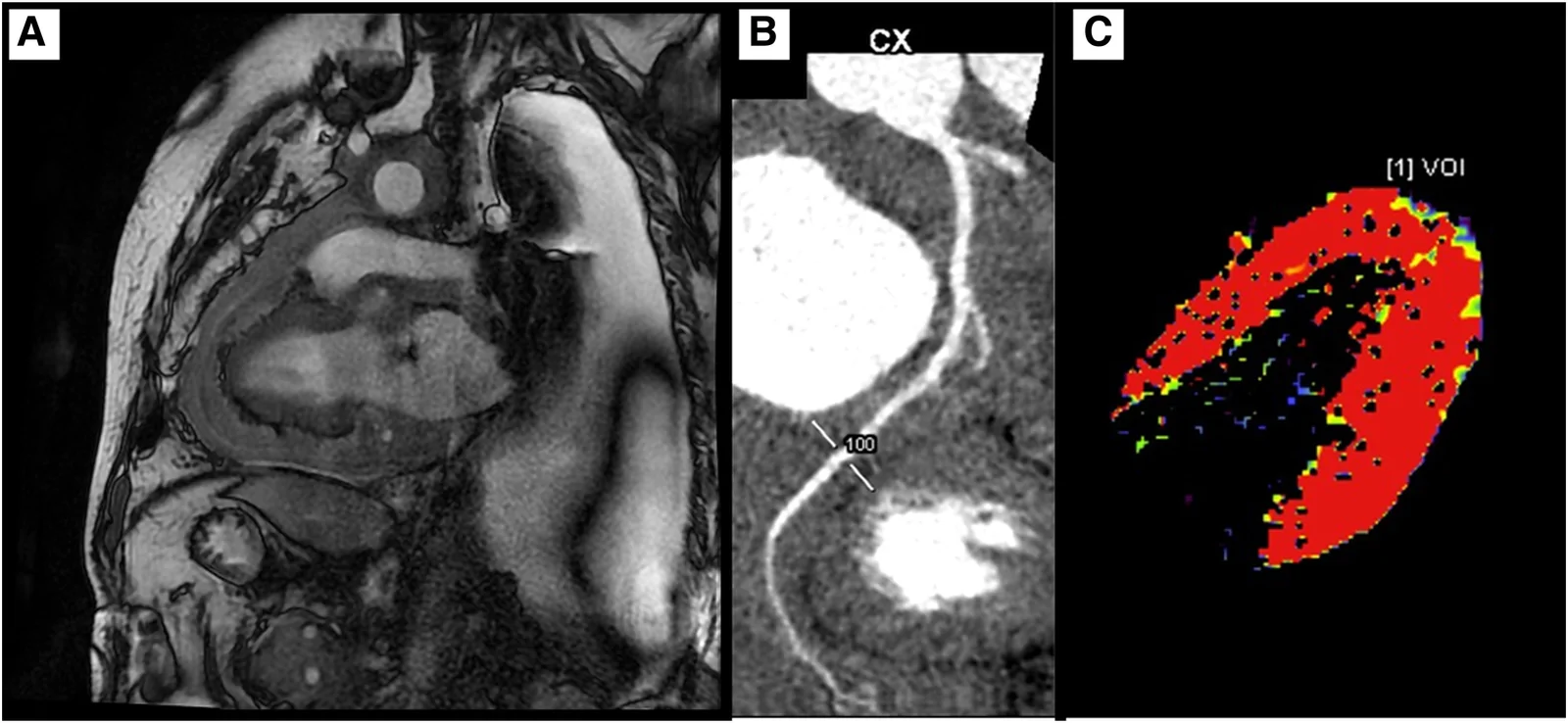
Cardiovascular magnetic resonance, computed tomography angiography, and myocardial perfusion imaging. Cardiovascular magnetic resonance demonstrated marked thickening of the pericardial layers, measuring up to 9–10 mm per layer, with pronounced post-contrast enhancement and a residual fluid layer of up to 4.6 mm between the pericardial layers (*A*). No significant coronary artery stenosis was identified on computed tomography angiography (*B*). No mismatch between myocardial blood flow (MBF) and myocardial blood volume (MBV) was observed (*C*).

Cardiac magnetic resonance demonstrated diffuse circumferential pericardial thickening of up to 10 mm per layer with marked late gadolinium enhancement (*Figure 3A*). Coronary computed tomography angiography excluded significant coronary artery stenosis but demonstrated circumferential perivascular soft-tissue thickening along the left main, left anterior descending, left circumflex, and right coronary arteries, as well as the ascending aorta, producing the characteristic ‘coated aorta’ appearance of ECD (*Figure 3B*). Computed tomography perfusion imaging showed no mismatch between myocardial blood flow and myocardial blood volume (*Figure 3C*).

The patient was treated with intravenous diuretics, systemic and topical antibiotics for superimposed skin infection, hormone replacement therapy, and glucocorticoids. This resulted in complete resolution of peripheral oedema, healing of the cutaneous lesions, reduction of pleural effusions, and substantial improvement in inflammatory markers, with a decrease in C-reactive protein to 37 mg/L and more than a two-fold reduction in NT-proBNP to 6504 pg/mL. Despite this initial clinical improvement, constrictive physiology persisted. Following multidisciplinary discussion involving cardiologists, cardiac surgeons, and haematologists, urgent pericardiectomy was considered the only feasible therapeutic option for constrictive hemodynamics.^10^ A subsequent disease-specific therapy for ECD was planned after surgical recovery. Intraoperatively, the pericardium was markedly thickened, densely adherent, and extended into the subepicardial tissue. Pericardiectomy was performed over the anterior, lateral, and basal cardiac surfaces.

The post-operative course was complicated by refractory cardiogenic shock and progressive multiorgan failure requiring high-dose inotropic support and mechanical ventilation. The patient failed to regain consciousness and died in the early post-operative period.

**Figure 4:**
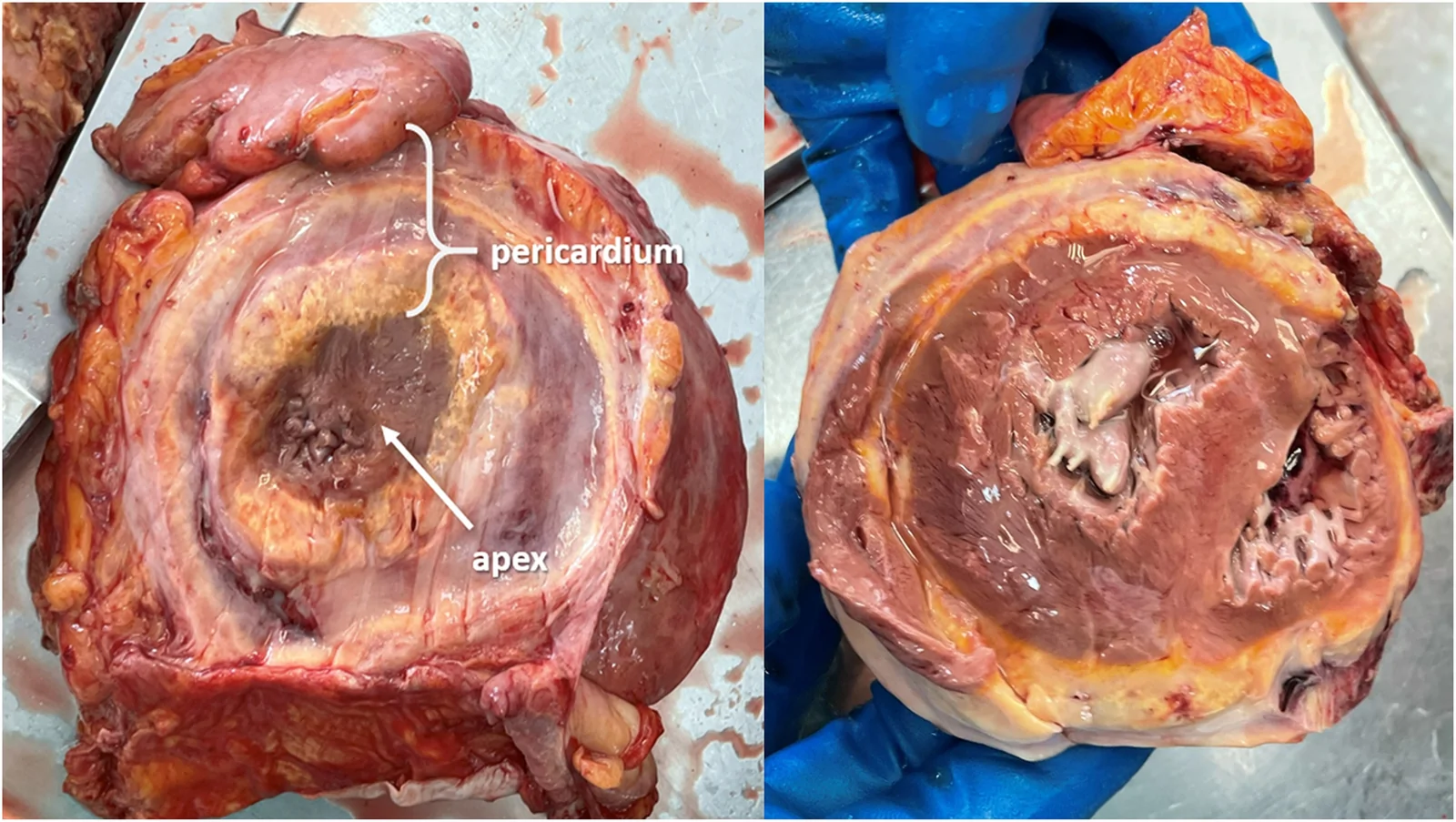
Autopsy findings. Gross examination of the heart revealed marked diffuse pericardial thickening with a greyish-white appearance. Portions of the pericardium had been surgically resected from the apical, anterior, lateral, and partially diaphragmatic regions. The heart was enlarged and dilated, with a total weight of 1120 g. On sectioning, the myocardium was reddish-brown with a firm-elastic consistency and scattered greyish-white streaks measuring 1–2 mm, with subepicardial extension of tumour infiltration.

**Figure 5:**
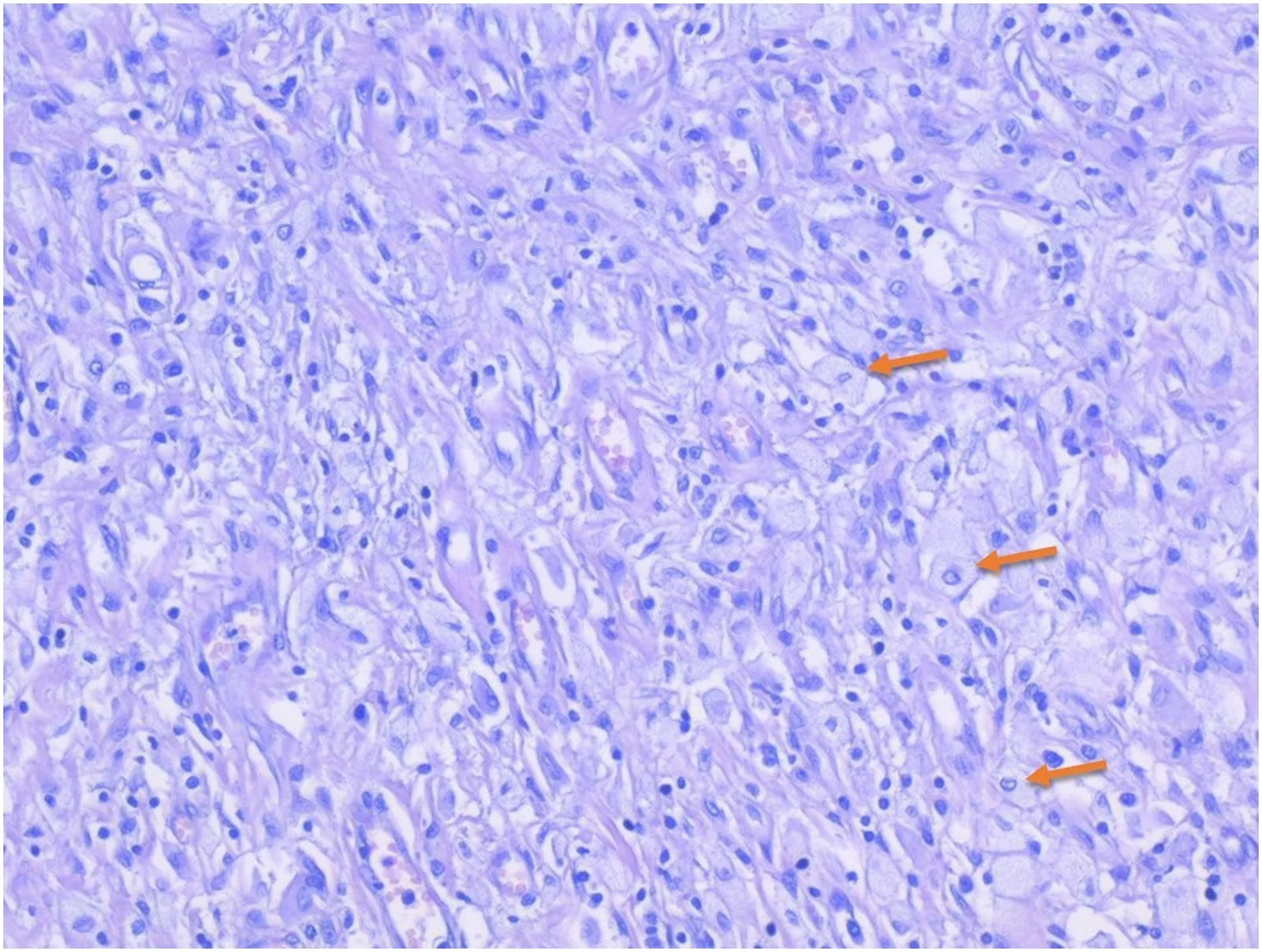
Myocardial histological findings. Photomicrograph of a sclerosed myocardial area showing infiltration by foamy histiocytes (arrows) intermixed with lymphocytes (haematoxylin and eosin staining, ×200).

Autopsy revealed extensive multisystem histiocytic infiltration, most pronounced within the cardiovascular system. Residual pericardial tissue demonstrated diffuse infiltration by foamy histiocytes with marked thickening (*Figure 4*). The myocardium contained widespread grey-white streak-like infiltrates measuring 1–2 mm, consistent with diffuse histiocytic myocardial infiltration (*Figure 5*). These pathological findings established that the patient’s constrictive physiology reflected advanced diffuse cardiovascular involvement rather than isolated constrictive pericardial disease, providing a pathological explanation for the unfavourable outcome despite technically successful pericardiectomy.

## Discussion

Erdheim–Chester disease is a rare clonal non-Langerhans cell histiocytosis characterized by multisystem infiltration of tissues by CD68-positive, CD1a-negative histiocytes.^2^ Although virtually any organ may be affected, prognosis is largely determined by the extent of involvement of the cardiovascular system, central nervous system, lungs, and retroperitoneum.^1,2^ Cardiovascular manifestations are reported in ∼40%–70% of patients when assessed by contemporary imaging modalities and represent one of the leading causes of disease-related mortality.^1,11,12^ Most patients remain asymptomatic until advanced stages, underscoring the importance of systematic cardiovascular evaluation at diagnosis and during follow-up.^2^

The spectrum of cardiovascular involvement in ECD is broad and includes circumferential peri-aortic and peri-coronary infiltration (‘coated artery’ signs), right atrioventricular pseudotumours, myocardial infiltration, valvular abnormalities, and pericardial disease.^1–4^ While pericardial thickening and effusion are relatively common imaging findings, progression to constrictive pericarditis is exceptionally rare, with fewer than 10 well-documented cases reported.^5–9^ Consequently, the optimal management of constrictive physiology in ECD remains undefined, and current recommendations are based almost exclusively on expert consensus and individual case reports.

The therapeutic management of advanced cardiac ECD remains challenging. Since the discovery of recurrent MAPK pathway alterations, ECD has been reclassified as a clonal myeloid neoplasm, fundamentally changing treatment strategies.^2^ Targeted therapy with BRAF or MAPK inhibitors has dramatically improved clinical outcomes and is currently considered the cornerstone of systemic treatment.^2^ That improved patients’ overall 5-year survival to 82.7% during the last two decades.^13^ In contrast, corticosteroids alone provide only transient symptomatic benefit and do not modify disease progression.^2^

The present case illustrates several important clinical aspects. First, constrictive pericarditis was the dominant manifestation of untreated progressive cardiovascular involvement in ECD, leading to advanced congestive heart failure. Second, multimodality imaging provided complementary information regarding both the haemodynamic consequences and the anatomical extent of the disease. Third, findings from autopsy with extensive myocardial infiltration explained the mechanism underlying refractory heart failure and the poor post-operative outcome despite technically successful pericardiectomy.

The presented patient was negative for BRAF mutation. The targeted therapies were not reimbursed for ECD within the national healthcare system during the patient’s treatment. This case therefore also illustrates the challenges of managing rare diseases in healthcare settings where advanced targeted treatments remain inaccessible.

## Lead author biography


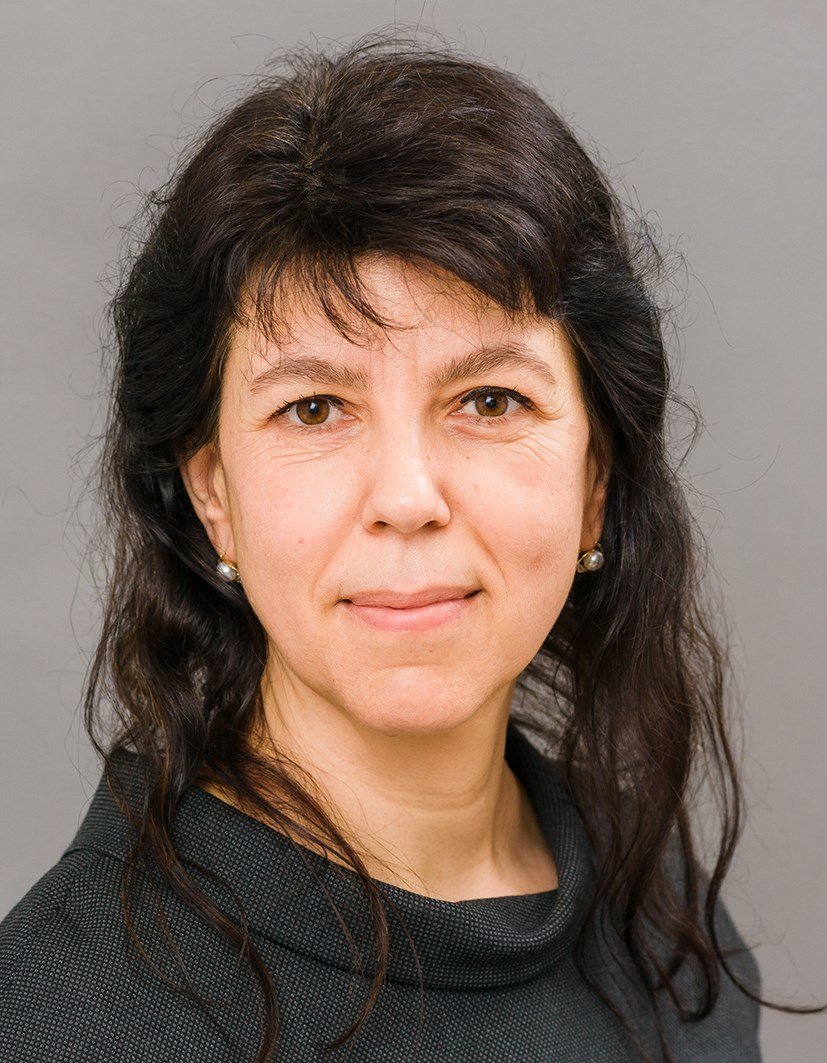
Assoc. Prof. M. Dimova-Mileva is a specialist in Internal Medicine and Cardiology, with additional ESC/EACVI certification in transthoracic echocardiography and expertise in abdominal and vascular ultrasonography.

## Data Availability

All data relevant to this case report are included in the manuscript and its figures. No additional datasets were generated or analysed.
